# Synthesis route-dependent structural and electrochemical properties of La_0.63_Ga_0.37_Mo_0.74_Fe_0.26_O_3−δ_ symmetrical electrodes for intermediate-temperature SOFCs

**DOI:** 10.1039/d6ra09159h

**Published:** 2026-11-12

**Authors:** Hamza Gulzar, M. Wasif Ghauri, M. Shakir Shareef, Seharish Javed, M. Ashfaq Ahmad, M. Ajmal Khan, Imran Shakir, Abdullah Almohammedi, Rizwan Raza, Ghazanfar Abbas

**Affiliations:** a Clean Energy Research Lab. (CERL), Department of Physics, COMSATS University Islamabad Lahore Campus 54000 Lahore Pakistan mian_ghazanfar@hotmail.com; b Deparment of Physics, Faculty of Science, Islamic University of Madinah Madinah 42351 Saudi Arabia

## Abstract

This study carefully explores the impact of synthesis route on the structural, optical and electrochemical properties of La_0.63_Ga_0.37_Mo_0.74_Fe_0.26_O_3−δ_ (LGMF) as a symmetrical electrode for intermediate temperature solid oxide fuel cells (IT-SOFCs). The LGMF material was synthesized *via* four distinct routes such as sol–gel, hydrothermal, co-precipitation and solid-state methods. The detailed characterization of the prepared materials revealed strong dependence of phase formation route, crystallinity and band gap. X-ray diffraction (XRD) and Raman spectra confirmed the formation of crystalline perovskite-related phases, with the co-precipitation and sol gel routes exhibiting improved crystallinity and structural homogeneity. UV-visible analysis shows a gradual reduction in band gap, narrowing from 2.26 eV to 1.93 eV indicating enhanced defect concentration and charge transport characteristics. Among all synthesis routes the co-precipitation method exhibits the lowest ohmic resistance as 0.44 Ω and the highest total conductivity of 0.70 S cm^−1^ at 650 °C (operating temperature). Consequently, fuel cell testing reveals that the co-precipitation-synthesized electrode has the highest fuel cell performance with a peak power density of 286 mW cm^−2^. The results prove that the co-precipitation method offers a suitable approach for preparing high-performance and redox-stable symmetrical electrodes for IT-SOFCs by improving electrocatalytic activity and ionic/electronic transport properties.

## Abstract 2

This study carefully explores the impact of synthesis routes on the structural, optical and electrochemical properties of La_0.63_Ga_0.37_Mo_0.74_Fe_0.26_O_3−δ_ (LGMF) as a symmetrical electrode for intermediate temperature solid oxide fuel cells (IT-SOFCs).

## Graphical abstract

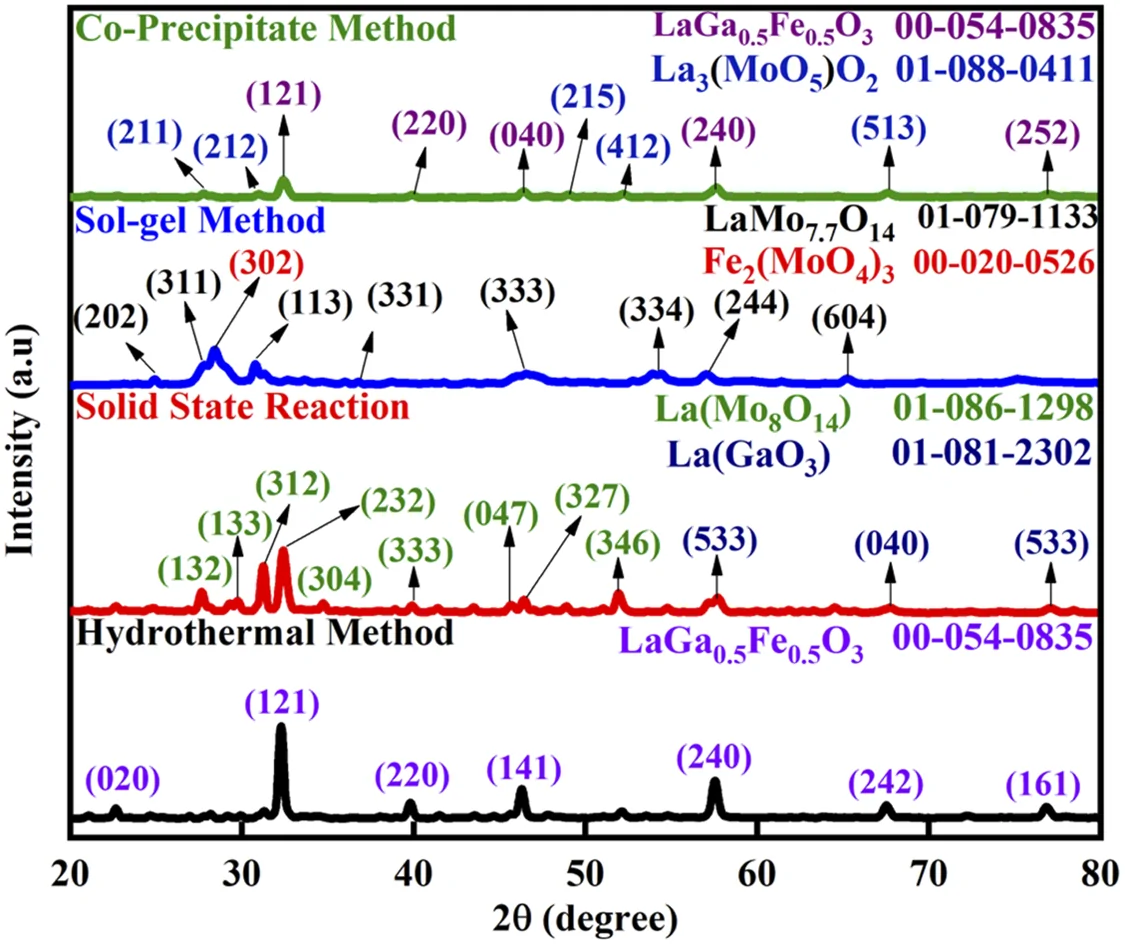

## 1. Introduction

Solid oxide fuel cells (SOFCs) have great potential that is underutilized especially considering the energy crisis and their benefit for environmentally friendly energy generation.^1–3^ SOFCs convert chemical energy into electrical energy for achieving efficiencies of up to 70% with zero emissions. Recent advances have focused on developing low- and intermediate-temperature SOFCs operating at 500–800 °C, as lowering the operating temperature can suppress component degradation, expand the range of suitable materials and reduce cost; however, it also decreases electrode kinetics and increases interfacial polarization resistance.^4^ Since SOFCs have excellent power efficiencies and fuel flexibility therefore, they are widely regarded as promising energy conversion devices for energy conversion technology.^5–10^ These key features make them suitable for the transition to non-conventional energy sources as they can produce the same energy output as traditional energy sources *e.g.*, generators while using much less fuel. Among other fuels, hydrogen is an ideal fuel for this technology which produces only water as a byproduct.^11,12^ In symmetrical SOFCs, the same material can be used for both anode and cathode with advantages such as simplified fabrication, resistance to carbon deposition and sulfur poisoning, and reduced interfacial diffusion issues.^13,14^ Symmetrical SOFCs based on perovskite electrodes have demonstrated promising electrochemical performance, with Ni-doped La_0.6_Sr_0.4_FeO_3−δ_ symmetrical electrodes achieving high open-circuit voltages and peak power densities under hydrocarbon-fuel conditions, together with good long-term stability.^15^

However, the electrode materials in SSOFCs are more rigorous, as they must perform reliably under both reducing and oxidizing environments. Perovskite-type oxides have attracted considerable attention for energy conversion and storage applications because of their tunable structure, defect chemistry and transport properties, particularly their mixed electronic and ionic conductivity, which is beneficial for electrochemical applications such as SOFCs.^16^

These electrodes must exhibit chemical and structural stability, along with excellent electrocatalytic activity to facilitate both fuel oxidation and oxygen reduction reaction.^17^ Numerous techniques have been used to improve conventional electrodes for increased stability and performance. Chen *et al.* developed a novel electrode architecture using La_0.7_Sr_0.3_Fe_0.7_Ga_0.3_O_3−δ_ (LSFG), which significantly reached at high power density of 489 mW cm^−2^ at 800 °C in a hydrogen (H_2_) environment, enhanced resistance to carbon deposition while maintaining excellent electrochemical performance under fuel-rich conditions.^18^ Ding *et al.* demonstrated that surface modification through infiltration of Pr_0.8_Sr_0.2_MnO_3−δ_ (PSM) and Pr_0.8_Sr_0.2_Co_0.2_Mn_0.8_O_3−δ_ (PSCM), significantly improved the electrocatalytic activity of La_0.6_Sr_0.4_Co_0.2_Fe_0.8_O_3−δ_ (LSCF) cathodes, enhancing their performance, modified cathode achieved OCV values of 1.1 V and 1.0 V with maximum power densities of 1.02 and 1.40 W cm^−2^ under SOFC operating conditions respectively.^19,20^ However, a few studies have systematically investigated the impact of synthesis methods on the redox stability and performance of Mo-substituted Perovskite in symmetrical configurations. Cai *et al.* studied La_0.5_Ba_0.5_Fe_0.95_Mo_0.05_O_3−δ_ symmetric electrodes and evaluated their electrochemical performance and stability under SOFC operating conditions.^21^ Bian *et al.* investigated La_0.5_Sr_0.5_Fe_0.9_Mo_0.1_O_3−δ_ electrodes for reversible symmetric solid oxide cells and demonstrated good redox stability and electrochemical performance.^22^ Shen *et al.* investigated Mo-doped Ba_0.5_Sr_0.5_Co_0.8_Fe_0.2_O_3−δ_ as a symmetric electrode and reported a maximum power density of 1225 mW cm^−2^ at 800 °C with wet H_2_, along with improved redox stability and thermal expansion compatibility.^23^ Cavazzani *et al.* reported that MnO_*x*_-impregnated La_0.6_Sr_0.4_Ga_0.3_Fe_0.7_O_3_ anodes achieved 70 mW cm^−2^ in H_2_ and 67 mW cm^−2^ in C_3_H_8_ at 744 °C.^24^ However, the electrochemical performance of LSGF-based symmetrical electrodes remains lower than that of conventional SOFC electrodes. The need to enhance the performance of electrodes for SSOFC and it can be boosted by the partial substitution of Fe by Mo in this study aims to modify the defect chemistry and enhance phase stability, leveraging the favourable electrochemical framework established while Fe contributes to mixed ionic-electronic conductivity *via* Fe^3+^/Fe^4+^ redox transitions, favouring defect-mediated transport for both oxygen and hydrogen electrochemical reactions by LSGF and by inclusion of Ga which promotes structural stability in reducing conditions. Prior investigations on LSGF-based electrodes demonstrate redox reversibility, compatibility with LSGM electrolytes, and excellent stability in reversible SOFCs and solid oxide electrolytic cells (SOECs) operation modes.^25–27^ In this study, La_0.63_Ga_0.37_Mo_0.74_Fe_0.26_O_3−δ_ was synthesized using four methods: sol–gel, hydrothermal, coprecipitation, and solid state reaction to evaluate the influence of synthesis route on its functional properties and their characterizations were analyzed like XRD, Raman, UV-vis, EIS, conductivity measurements and fuel cell performances.

## 2. Experimental

### 2.1. Synthesis of electrode and electrolyte materials

The following chemicals of lanthanum(iii) nitrate hexahydrate La(NO_3_)_3_·6H_2_O (Sigma-Aldrich, 99.99%), gallium(iii) nitrate hydrate (Ga(NO_3_)_3_·*x*H_2_O) (Macklin, 99.9%), iron(iii) nitrate nonahydrate (Fe(NO_3_)_3_·9H_2_O) (Sigma-Aldrich, ≥ 99.95%) and molybdenum(vi) oxide (MoO_3_) (nanochemazone, 99%) were used as precursor materials with a stoichiometric ratio of La_0.63_Ga_0.37_Mo_0.74_Fe_0.26_O_3−δ_ (LGMF). Stoichiometric ratio of citric acid monohydrate (C_6_H_8_O_7_·H_2_O) (Samchun, 99.5%) 5 : 1 (LGMF : citric acid) and sodium carbonate anhydrous (Na_2_CO_3_) (Sigma-Aldrich, 99%) 1 : 2 (LGMF : Na_2_CO_3_) were taken into account as chelating and precipitating agents for sol–gel and co-precipitation methods, respectively. Four samples were synthesized by sol–gel method, hydrothermal process, co-precipitation method and solid-state reaction. In the hydrothermal method, aqueous precursor solution was treated in a Teflon-lined autoclave at 200 °C for 12 hours, where crystallization began and nanoparticles grew *in situ* and the product was filter and washed until pH ≈ 10 reached. In the solid-state reaction method, the stoichiometric powders were thoroughly mixed, ground using an agate mortar and pestle to ensure homogeneity. In the sol–gel, metal nitrates were dissolved in deionized water (DIW), the polymerized gel was formed by addition of citric acid (C_6_H_8_O_7_). In the co-precipitation, metal nitrates were dissolved in water and precipitates were obtained by adding the aqueous solution of sodium carbonate (Na_2_CO_3_) dropwise. The precipitates were obtained by filtration and washing. All the precursors obtained by these four synthesis routes were dried at 200 °C in an oven for 6 hours followed by sintering at 900 °C for 4 hours in digital furnace. The sintered materials were annealed and ground again. The GDC electrolyte material was synthesized using co-precipitation. The detailed preparation method is reported in published article.^28^

### 2.2. Fuel cell fabrication

In order to obtain the mixed ionic and electronic (MIEC) conduction in the prepared electrodes, the composites contained 80 wt% LGMF (electrode) – 20 wt% GDC (electrolyte). The three layers were fabricated having the cell scheme as LGMF–GDC (anode)/GDC (electrolyte)/LGMF–GDC (cathode) for all types of synthesized materials. The cells were fabricated with a diameter of 13 mm and 1 mm thick (set as anode (0.40 mm), electrolyte (0.35 mm) and cathode (0.25 mm) respectively) using hydraulic press with pressure of 300 kg cm^−2^. All cells were sintered at 800 °C for 60 minutes, both surfaces of the cells anode and cathode were painted with silver paste to serve as a current collector.

### 2.3. Characterization

The synthesized LGMF electrode materials were successfully characterized by XRD, Raman, UV-vis, EIS, conductivity measurements and fuel cell performances. The D/Max-3A Regaku 6100 X-ray diffractometer using Cu Kα radiation (*λ* = 1.5418 Å) at a voltage of 35 kilovolts and a current of 30 mA was used to obtain the XRD patterns. The vibrational modes and local structure were investigated with Raman microscope by RENISHAW UK using laser excitation at 488 nm. The UV/vis/NIR spectrophotometer (PerkinElmer LAMBDA 750) was used to analyse the absorption spectrum of LGMF electrode within the 200–800 nm range. Electrochemical impedance spectroscopy was achieved on three-layer fabricated cells, using an electrochemical workstation (CorrTest CS350) measures the materials' internal resistance and computes the conductivity values using the formula in eqn (1).1
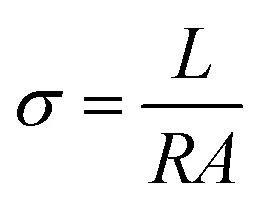
where *σ* shows conductivity, *R* represents ohmic resistance, *A* is an active area of fabricated cell which is 0.64 cm^2^ and *L* is the thickness of the cell (1 mm). Fuel cell testing unit (SM-102, China) was used to measure fuel cell's performance at 650 °C with flow rate of 100 ml min^−1^ hydrogen at the anode side and 80 ml min^−1^ air at the cathode side.

## 3. Results and discussion

### 3.1. Structural properties of LGMF materials

The X-ray diffraction (XRD) patterns of La_0.63_Ga_0.37_Mo_0.74_Fe_0.26_O_3−δ_ were obtained to compare the crystalline phases synthesized by hydrothermal, solid-state reaction, sol–gel and co-precipitation methods, where each method shows distinct diffraction peaks with their respective Miller indices, measured 2*θ* positions and matched JCPDS card numbers for phase identification as summarized in Table 1.

**Table 1:** XRD phase identification and crystal structure parameters for LGMF cathode materials

Synthesis method	Observedplanes (*hkl*)	Identifiedphase	JCPDScard no.	Crystalsystem	Phase purity/Segregation
Hydrothermal	(020), (121), (220), (141), (240), (242), (161)	LaGa_0.5_Fe_0.5_O_3_	00-054-0835	Orthorhombic	Pure single-phase
Solid-state	(132), (133), (312), (232), (304), (333), (040), (533)	LaMo_8_O_14_/LaGaO_3_	01-086-1298/01-081-2302	Orthorhombic	Mixed phase
Sol–gel	(202), (311), (113), (331), (333), (334), (244), (604), (302)	LaMo_7.7_O_14_/Fe_2_(MoO_4_)_3_	01-079-1133/00-020-0526	Orthorhombic	Mixed phase
Co-precipitation	(211), (212), (215), (412), (513), (121), (220), (040), (240), (252)	La_3_(MoO_5_)O_2_/LaGa_0.5_Fe_0.5_O_3_	01-088-0411/00-054-0835	Orthorhombic	Mixed phase

In the hydrothermal method, peaks such as (020), (121), (220), (141), (240), (242) and (161) correspond to LaGa_0.5_Fe_0.5_O_3_ (JCPDS 00-054-0835) with orthorhombic structure, suggesting the presence of a single oxide phase. For the solid-state reaction, reflections at (132), (133), (312), (232), (304), (333), (047), (327), (346), (533), (040) and (533) are indexed to LaMo_8_O_14_ (JCPDS 01-086-1298) with orthorhombic structure and LaGaO_3_ (JCPDS 01-081-2302) with orthorhombic structure indicating mixed lanthanum molybdate and lanthanum gallate phases. The sol–gel method exhibits strong peaks at (202), (311), (113), (331), (333), (334), (244) and (604) which match those of LaMo_7.7_O_14_ (JCPDS 01-079-1133) with orthorhombic structure and peak at (302) which match those of Fe_2_(MoO_4_)_3_ (JCPDS 00-020-0526) with orthorhombic structure suggesting improved crystallinity and homogeneous phase formation due to molecular-level mixing of precursors. Finally, in the co-precipitation method, peaks such as (211), (212), (215), (412) and (513) are consistent with La_3_(MoO_5_)O_2_ (JCPDS 01-088-0411) with an orthorhombic structure, while peaks such as (121), (220), (040), (240) and (252) are consistent with LaGa_0.5_Fe_0.5_O_3_ (JCPDS 00-054-0835) with an orthorhombic structure, indicating mixed lanthanum molybdate and lanthanum gallate phases shown in Fig. 1.

**Fig. 1:**
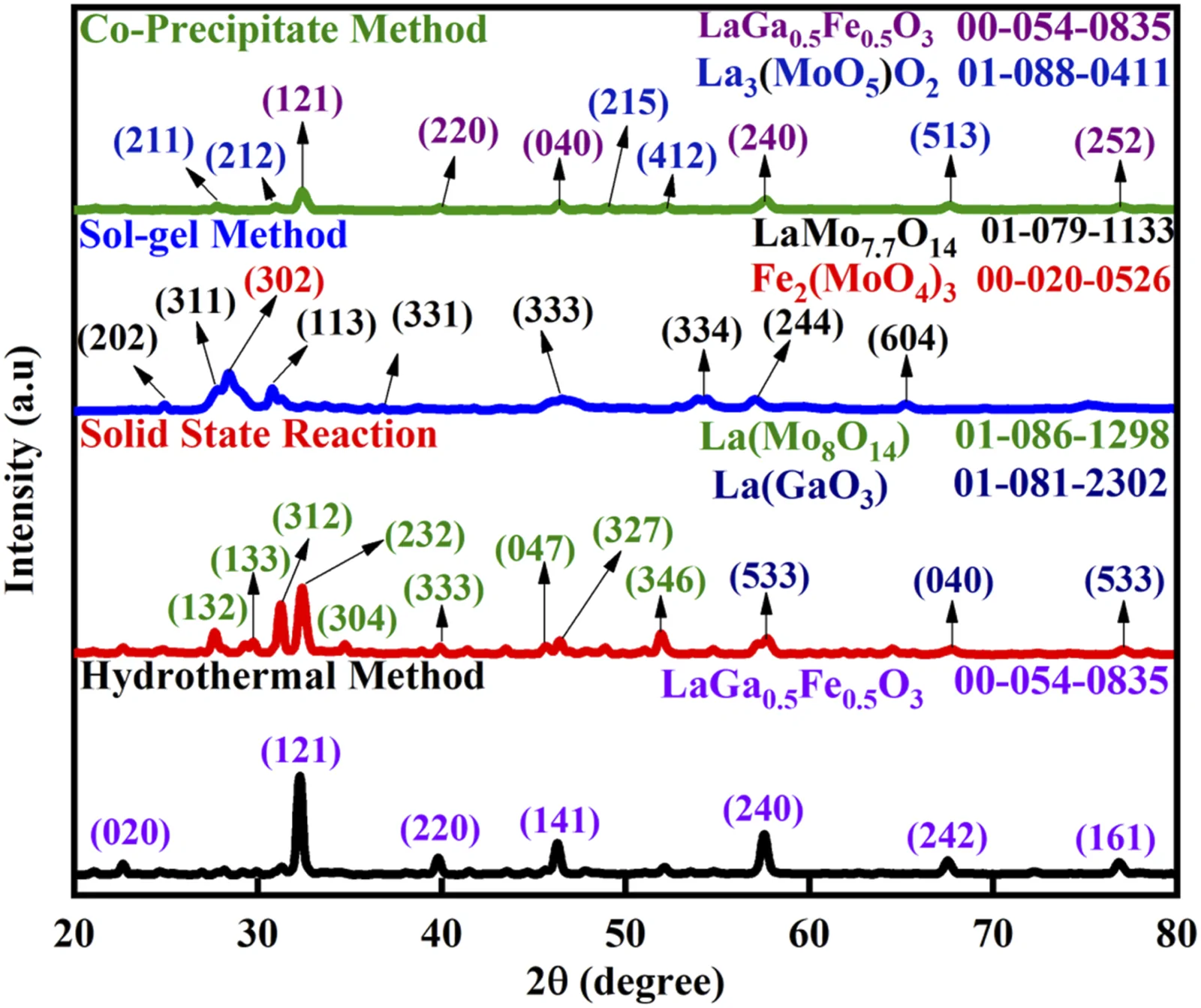
XRD patterns of La_0.63_Ga_0.37_Mo_0.74_Fe_0.26_O_3−δ_ (LGMF) samples synthesized *via* hydrothermal, solid-state, sol–gel and co-precipitation methods.

To quantitatively evaluate the microstructural evolution, lattice distortion and defects resulting from the different synthesis routes, key structural parameters were calculated using standard crystallographic relations. The inter-planar *d*-spacing and lattice constants (*a*, *b*, *c*) for the orthorhombic phase and unit cell volume were calculated using eqn (2) and (3):2
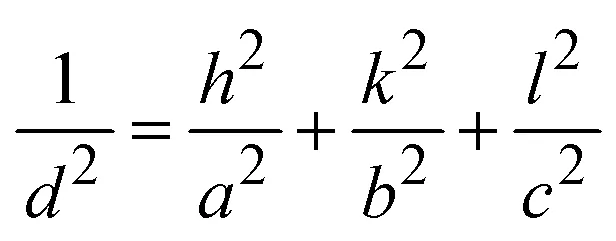

3 *V* = *a* × *b* × *c*

The average crystallite size (*D*) was determined from the full width at half-maximum *β* of the most prominent diffraction peak using Scherrer's equation (eqn (4)):4
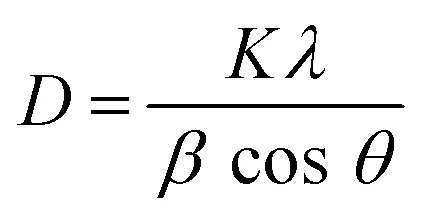
where *K* is the shape factor (0.9), *λ* is the X-ray wavelength (1.5406 Å for Cu-Kα radiation), *β* is the structural line broadening in radians and *θ* is the Bragg diffraction angle. Furthermore, lattice strain (*ε*) and dislocation density (*δ*) were calculated using the Stokes-Wilson and Williamson–Smallman equations, respectively (eqn (5) and (6)):5
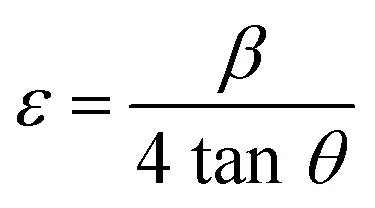
6
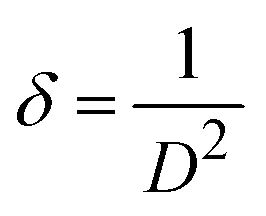


The complete calculated structural metrics are summarized in Table 2.

**Table 2:** Calculated structural parameters, lattice constants, unit-cell volume, strain and dislocation density

Synthesis method	Primary phase	*a* (Å)	*b* (Å)	*c* (Å)	*V* (Å^3^)	*D* (nm)	Strain *ε* × 10^−3^	*δ* × 10^14^ m^−2^ lines per m^2^
Hydrothermal	LaGa_0.5_Fe_0.5_O_3_	5.534	7.824	5.538	239.85	19.86	1.8	0.0026
Solid-state	LaMo_8_O_14_/LaGaO_3_	9.2065/5.529	11.1298/5.494	20.0264/7.785	2052.04/236.54	18.4	2.1	0.0042
Sol–gel	LaMo_7.7_O_14_/Fe_2_(MoO_4_)_3_	11.170/12.760	9.984/9.140	9.196/9.200	1025.71/1072.96	17.27	3.9	0.0159
Co-precipitation	La_3_(MoO_5_)O_2_/LaGa_0.5_Fe_0.5_O_3_	7.597/5.534	7.719/7.824	11.095/5.538	650.66/239.85	21.02	1.8	0.0027

Overall, these results demonstrate that the choice of synthesis route significantly influences phase purity, lattice parameters, strain and crystallite size. Hydrothermal synthesis leads to a clean, single oxide phase with moderate crystallite size (19.86 nm) and low dislocation density. The solid-state reaction results in mixed lanthanum molybdate and gallate phases with substantial lattice distortion, high micro-strain (2.1 × 10^−3^) and smaller domain size (18.4 nm). The sol–gel method yields improved overall crystallinity, reduced micro-strain (3.9 × 10^−3^) and optimized crystallite growth (17.27 nm) due to homogeneous molecular precursor mixing. Finally, co-precipitation leads to mixed phases characterized by sharp, high-intensity diffraction reflections, intermediate crystallite size (21.02 nm) and low defect density, as illustrated in Fig. 1.

### 3.2. Raman analysis

The comparative Raman spectra reveals pronounced modifications in the vibrational dynamics of the lattice as a function of the synthesis route. Materials fabricated *via* the hydrothermal methodology exhibit highly resolved and sharp phonon modes, indicating improved crystallinity and vigorous metal–oxygen coordination environments. The co-precipitation route, however, induces a noticeable red-shifting of all principal vibrational band's concomitant with spectral broadening, indicative of enhanced phonon-defect scattering and local structural perturbations. Specifically, the La–O initially centered at ∼299 cm^−1^ in the hydrothermal specimen undergoes a downward frequency shift in the co-precipitated counterpart,^29^ while the Ga–O vibrational resonance near 497 cm^−1^ and the Ag1 modes positioned at ∼638 correspond to Fe–O symmetric stretching.^30,31^ The Raman band at approximately 805 cm^−1^ is attributed to Mo–O stretching vibrations, whereas the strong band at 886 cm^−1^ is assigned to the symmetric stretching mode, *ν*_1_ (*A*_g_), of [MoO_4_]^2−^ tetrahedral units, consistent with the assignment reported for SrMoO_4_ by Gancheva *et al.* (2025). Variations in Mo–O bond lengths and local tetrahedral symmetry can affect these vibrational frequencies. In the co-precipitation sample, the band shifts from 886 to approximately 880 cm^−1^, suggesting a change in the local bonding environment. Compared with the sharper bands of the hydrothermal sample, the broader bands observed for the co-precipitation sample indicate synthesis-route-dependent changes in local structural order and vibrational behaviour^32,33^ in Fig. 2.

**Fig. 2:**
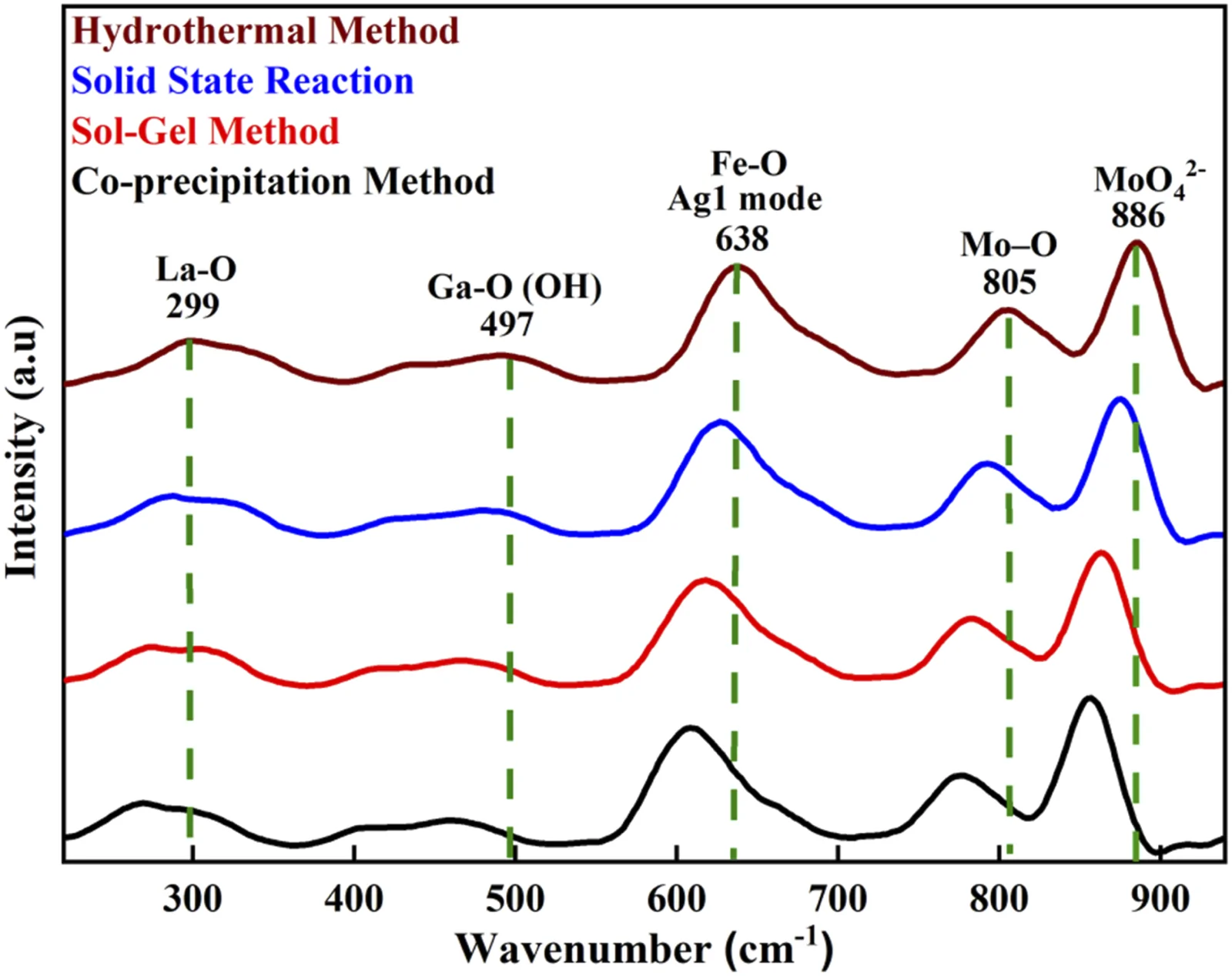
Raman spectra corresponding to different synthesis approaches for La_0.63_Ga_0.37_Mo_0.74_Fe_0.26_O_3−δ_ (LGMF).

### 3.3. UV-visible analysis

The wavelength and absorbance graphs show how each synthesis method shifts the absorption edge such as hydrothermal and solid-state reaction samples absorb strongly only at shorter wavelengths (UV region), while sol–gel and co-precipitation methods extend absorption further into the visible range. This red shift indicates a reduction in the band gap with increasing structural disorder and defect states.

The optical band gap was estimated from the Tauc's plots by extrapolating the linear region of (*αhν*)^2^ and *hν*. Hydrothermal synthesis yields the highest band gap (2.26 eV), reflecting better crystallinity and fewer defect states while solid-state method slightly lowers the band gap (2.1 eV) due to strain and grain boundary effects. Sol–gel (2.0 eV) and co-precipitation (1.93 eV) methods show further reduction in band gap, as they often produce materials with more porosity, disorder, and oxygen vacancies in Fig. 3. Thus, the optical analysis demonstrates how synthesis routes tune the band structure and influence the suitability of the material for SOFC performance.^34^ The reduced band gap observed for the co-precipitation sample indicates the presence of additional defect-related states within the electronic structure, which can facilitate charge-carrier excitation and transport. Such defect states and the associated reduction in band gap can influence the electronic transport behavior of the electrode, which is relevant to its electrochemical performance in SOFCs. By narrowing the band gap and introducing oxygen vacancies or structural disorder, the synthesis route directly tailors the material's conductivity and charge transfer kinetics at the electrode interface.^35^

**Fig. 3:**
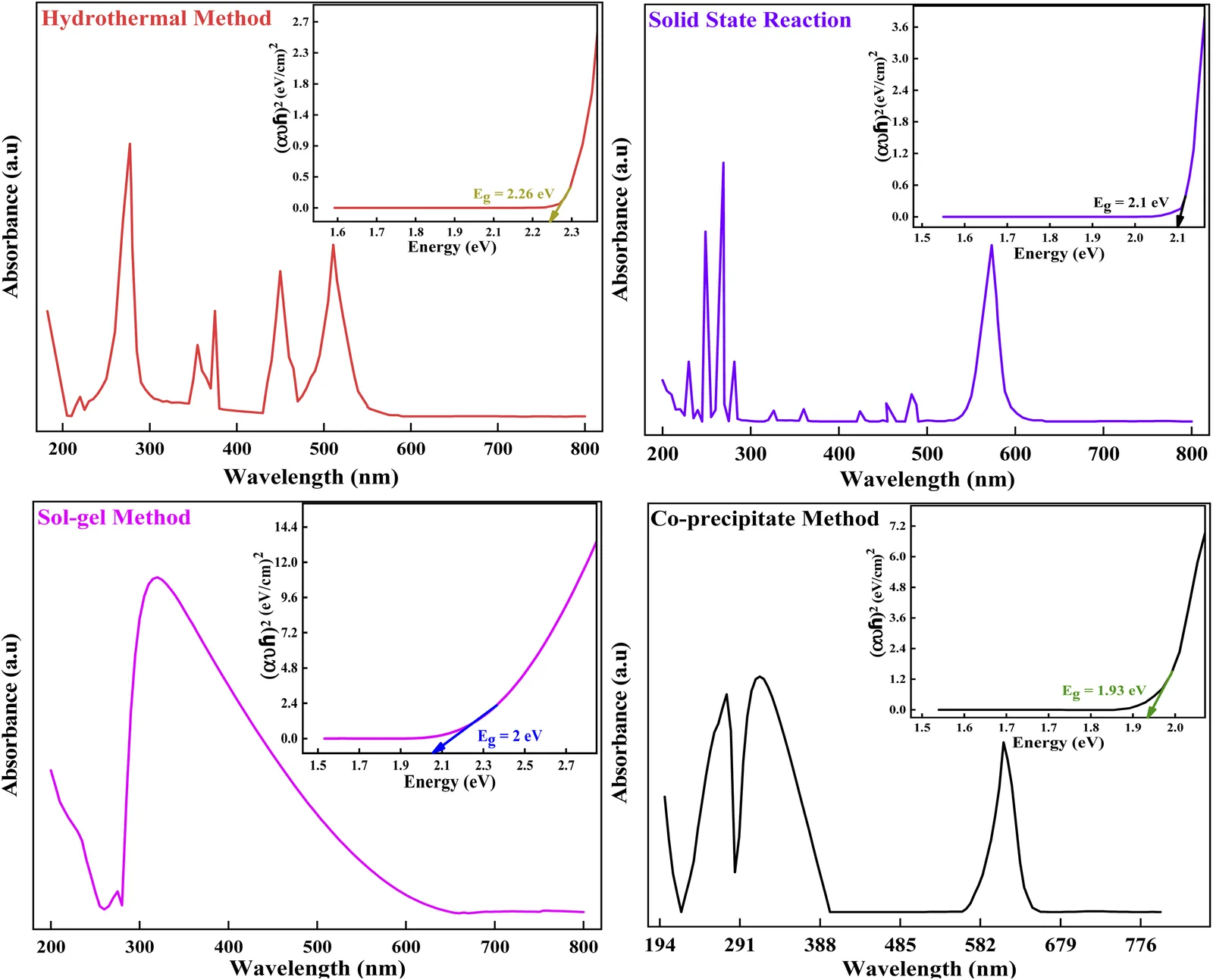
Effect of synthesis methods on UV-visible spectra and Tauc plots of La_0.63_Ga_0.37_Mo_0.74_Fe_0.26_O_3−δ_ (LGMF).

### 3.4. Electrochemical impedance spectroscopy (EIS) analysis

Electrochemical impedance spectroscopy (EIS) was employed to assess the electrochemical behavior of LGMF electrodes synthesized *via* co-precipitation, hydrothermal, sol–gel, and solid-state routes at intermediate temperatures (550–650 °C). The ohmic resistance (*R*_s_), obtained from the high-frequency intercept of the Nyquist plot on the real axis, was used as the primary metric for analyzing ionic conductivity and electrode performance. The results indicate a consistent decrease in *R*_s_ with increasing temperature for all synthesis methods, confirming the thermally activated nature of charge transport.

At 650 °C, the co-precipitation method achieved the lowest *R*_s_ value (0.44 Ω), suggesting enhanced ionic/electronic conductivity within the electrode. The solid-state route also demonstrated competitive performance at high temperature (0.72 Ω), indicating its potential for stable high-temperature operation. The sol–gel method exhibited a moderate *R*_s_ value of 0.57 Ω, whereas the hydrothermal method showed the highest *R*_s_ at 650 °C (0.85 Ω). Lower *R*_s_ values at higher temperatures indicate improved ionic/electronic conduction, which is critical for enhancing SOFC efficiency and reducing operational losses. At 600 °C, the co-precipitation, hydrothermal, sol–gel, and solid-state methods showed *R*_s_ values of 0.85 Ω, 1.61 Ω, 1.0 Ω, and 1.26 Ω, respectively, further demonstrating the trend of reduced resistance with increasing temperature. At 550 °C, all samples showed significantly higher *R*_s_ values, with the hydrothermal method reaching 2.34 Ω, the solid-state route 2.02 Ω, the sol–gel method 1.78 Ω, and the co-precipitation method 1.59 Ω, reflecting increased resistance and reduced conductivity at lower temperatures in Fig. 4(a–d). These observations underscore the influence of synthesis strategy on ohmic characteristics and overall electrode performance in solid oxide fuel cell applications. The EIS discussion has been extended by adding the linear fit curves using Z-View Software applying the equivalent model circuits displayed in the inset of Fig. 4(a–d). The EIS spectra of LGMF cathode synthesized by various synthesis route were recorded at 550, 600, and 600 °C in hydrogen/air environment to study the conduction mechanism of the materials. The electrochemical behavior is presented *via* Nyquist plots depicted in Fig. 4(a–d) showing the ionic as well as electronic behavior depending. The high-frequency region is generally associated with ohmic and charge-transfer processes, whereas the low-frequency response is related to surface adsorption, oxygen diffusion, and mass-transfer phenomena. Electrochemical impedance spectra behaviour displays the semicircles with low-frequency diffusion tails, indicating combined ionic and electronic conductivity.

**Fig. 4:**
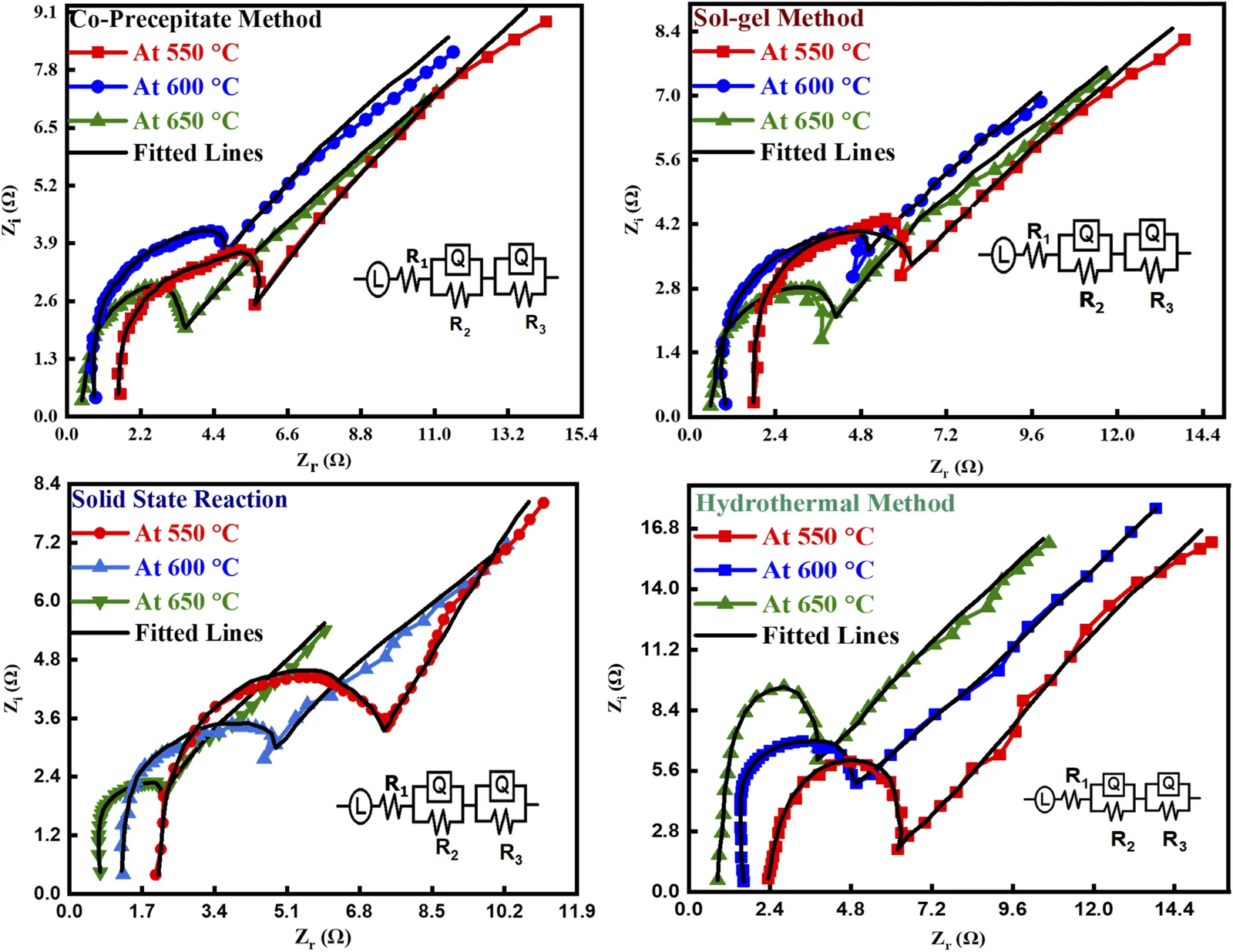
ElS spectra of La_0.63_Ga_0.37_Mo_0.74_Fe_0.26_O_3−δ_ (LGMF) cathode at 550–650 °C with various synthesis method.

### 3.5. Conductivity measurements

The graph presents the variation of conductivity with temperature for four different synthesis methods: co-precipitation, sol–gel, solid-state reaction, and hydrothermal. At 550 °C, the initial conductivity values are approximately 0.21 S cm^−1^ for the co-precipitation method, 0.18 S cm^−1^ for the sol–gel method, 0.17 S cm^−1^ for the solid-state reaction, and 0.15 S cm^−1^ for the hydrothermal method. When the temperature rises to 600 °C, conductivity increases due to enhanced thermal activation of charge carriers such as 0.40 S cm^−1^ (co-precipitation), 0.31 S cm^−1^ (sol–gel), 0.27 S cm^−1^ (solid-state reaction), and 0.20 S cm^−1^ (hydrothermal). The highest measured conductivity of La_0.63_Ga_0.37_Mo_0.74_Fe_0.26_O_3−δ_ (LGMF) cathode material at 650 °C reaches to 0.700 S cm^−1^ for co-precipitation, 0.531 S cm^−1^ for sol–gel, 0.428 S cm^−1^ for solid-state reaction, and 0.354 S cm^−1^ for hydrothermal. The results of measurements were displayed in Fig. 5(a). This trend demonstrates the thermally activated conduction behavior of the material, where electrical conductivity rises with temperature because more electrons or ions gain sufficient energy to overcome activation barriers, thereby increasing charge carrier density and mobility. The co-precipitation method yields the highest conductivity at every temperature, suggesting crystallinity, smaller particle size, and reduced defect density, which minimize scattering and grain boundary resistance. The sol–gel method ranks second, likely because it produces a moderately homogeneous microstructure, though still with more defects than co-precipitation. The solid-state reaction method results in lower conductivity, possibly due to larger crystallites and incomplete diffusion during synthesis, which limit carrier pathways. The hydrothermal method consistently shows the lowest values, implying that it may introduce porosity, structural disorder or secondary phases that act as charge-trapping sites. Thus, while all methods exhibit thermally activated conduction, the co-precipitation route is the most effective for achieving high conductivity, whereas the hydrothermal method is the least favorable for optimizing electronic transport properties. The reported conductivity is the total conductivity derived from the EIS ohmic resistance curves from all the samples. In this regard, the conductivity formula is given below:

7 *σ*_tot_ = *σ*_el_ + *σ*_io_

where *σ*_tot_ represents the total conductivity, *σ*_el_ denotes the electronic conductivity, and *σ*_io_ prescribes the ionic conductivity. In this measurement, the GDC electrolyte was used for all the samples, so the ionic conductivity of GDC electrolyte has been recorded 0.094 S cm^−1^. The incorporated and previous results have been shown in Table 3.

**Fig. 5:**
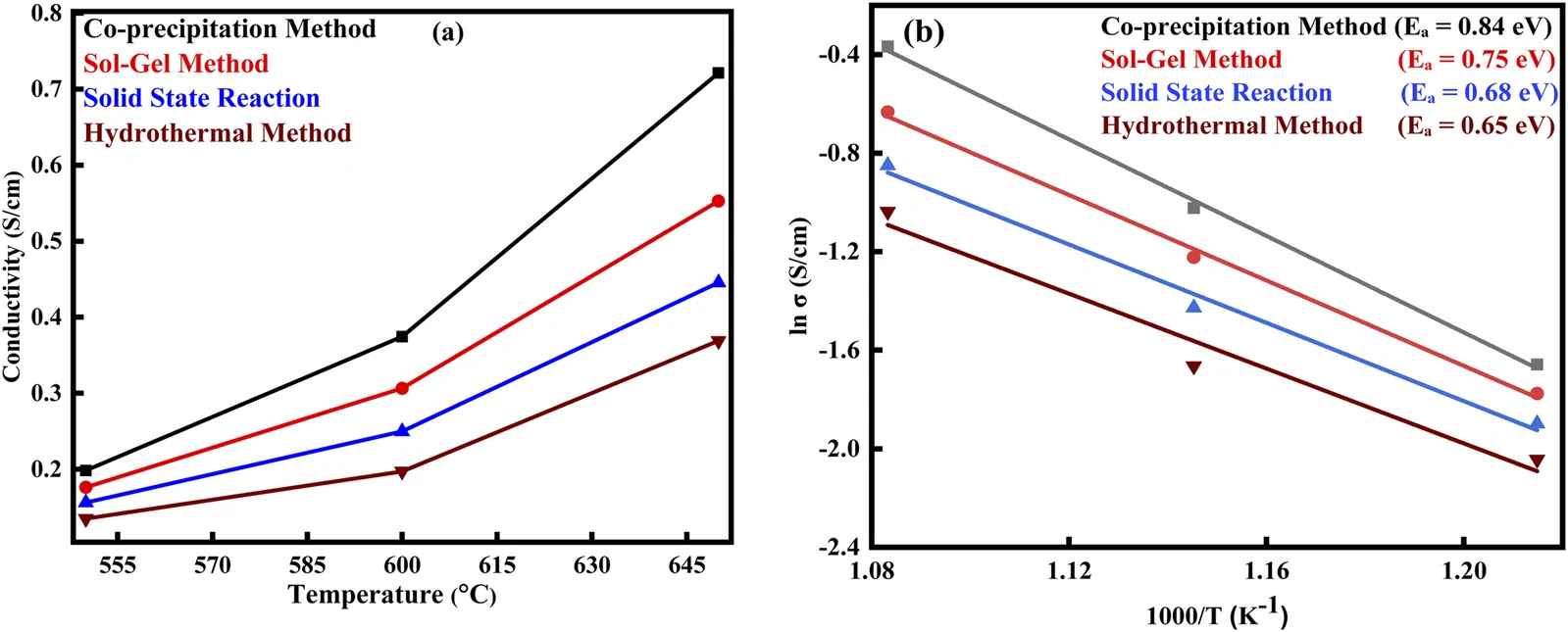
(a) Temperature-dependent electrical conductivity (b) temperature dependent activation energies of La_0.63_Ga_0.37_Mo_0.74_Fe_0.26_O_3−δ_ (LGMF) cathode material synthesized by co-precipitation, solid-state reaction, and hydrothermal routes.

**Table 3:** The conductivities, activation energies, and area specific resistance data of La_0.63_Ga_0.37_Mo_0.74_Fe_0.26_O_3−δ_ (LGMF) cathode material at 650 °C

Materialsynthesis route	Totalconductivity *σ*_tot_ (S cm^−1^)	Ionicconductivity *σ*_io_ (S cm^−1^)	Electronicconductivity *σ*_el_ (S cm^−1^)	Activation energy *E*_a_ (eV)	Area specific resistance ASR (Ω cm^2^)
Co-precipitation	0.700	0.094	0.606	0.840	0.282
Sol–gel method	0.531	0.094	0.437	0.750	0.641
Solid state reaction	0.428	0.094	0.334	0.680	0.463
Hydrothermal	0.354	0.094	0.260	0.650	0.544

The valuable addition of Area Specific Resistance ASR and activation energy measurements enables the electrochemical study more convenient to readers. The ASR has been calculated from resistivity data and activation energy graphs have been shown in Table 3 and Fig. 5(b). The activation energy of LGMF cathode materials synthesized *via* various synthesis routes for conduction has been obtained by plotting electrical conductivity data in Arrhenius relation in hydrogen atmosphere using formula below:8
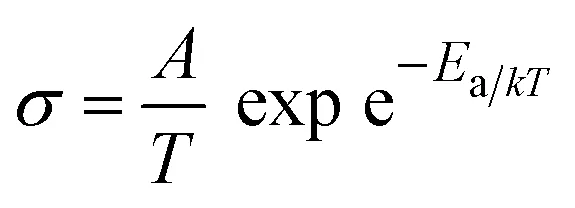
where *σ* is electrical conductivity, *T* is temperature in kelvin, *A* is the exponential factor, *k* is Boltzman's constant and *E*_a_ is the activation energy. The activation energy plays an important role in evaluating performance for oxygen diffusion and oxygen ion conductivity in cathode material. The oxygen reduction reaction ORR occurs at the cathode (where incoming oxygen molecules are reduced into oxygen ions) is predictably the primary rate-limiting step for overall SOFC performance.

### 3.6. Fuel cell performance

The Fig. 6(a) shows the current and power density curves of fuel cells operated at 650 °C with hydrogen as the fuel and air as the oxidant, synthesized by four different preparation methods such as Co-precipitation, Sol–Gel, solid-state reaction, and Hydrothermal. The *x*-axis shows the current density (mA cm^−2^), the left *y*-axis corresponds to the cell voltage (*V*), and the right *y*-axis represents the power density (mW cm^−2^). In all cases, the voltage decreases as current density increases due to activation, ohmic, and concentration losses, while the power density exhibits a parabolic shape, increasing to a maximum value before decreasing as mass transport limitations dominate. The co-precipitation method shows the most promising electrochemical performance, with the highest open-circuit voltage (OCV) of 0.94 V and a peak power density reaching around 286 mW cm^−2^ at a current density near 616 mA cm^−2^. This indicates excellent catalytic activity, better electrode–electrolyte interaction, and low internal resistance. The Sol–Gel method also performs well, showing an OCV of 0.89 V and a maximum power density of around 242 mW cm^−2^ at a current density close to 600 mA cm^−2^, slightly lower than co-precipitation but still efficient. The solid-state reaction method provides moderate performance, with an OCV of roughly 0.85 V and a maximum power density of 192 mW cm^−2^ occurring at 581 mA cm^−2^. This reflects higher ohmic losses compared to the first two methods. In contrast, the hydrothermal method delivers the weakest performance, with the lowest OCV of 0.81 V and the lowest maximum power density around 144 mW cm^−2^ at 534 mA cm^−2^, which suggests less effective catalytic behaviour. Overall, the data clearly demonstrate that the synthesis method plays a crucial role in determining the fuel cell's performance. The co-precipitation method achieves the highest efficiency and stability under hydrogen/air operation, followed by the sol–gel and solid-state reaction methods, while the hydrothermal method lags in both OCV and power output. This highlights that careful control of synthesis routes is essential to optimizing the electrochemical characteristics of fuel cells. The best performance of 286 mW cm^−2^ has been reported in the sample synthesized by co-precipitation route. So, the stability has been observed of the same sample for 24 hours and the stability test has been displayed in Fig. 6(b). It has been noted that stability is approximately constant with minor degradation of 0.94 V to 0.8901 V.

**Fig. 6:**
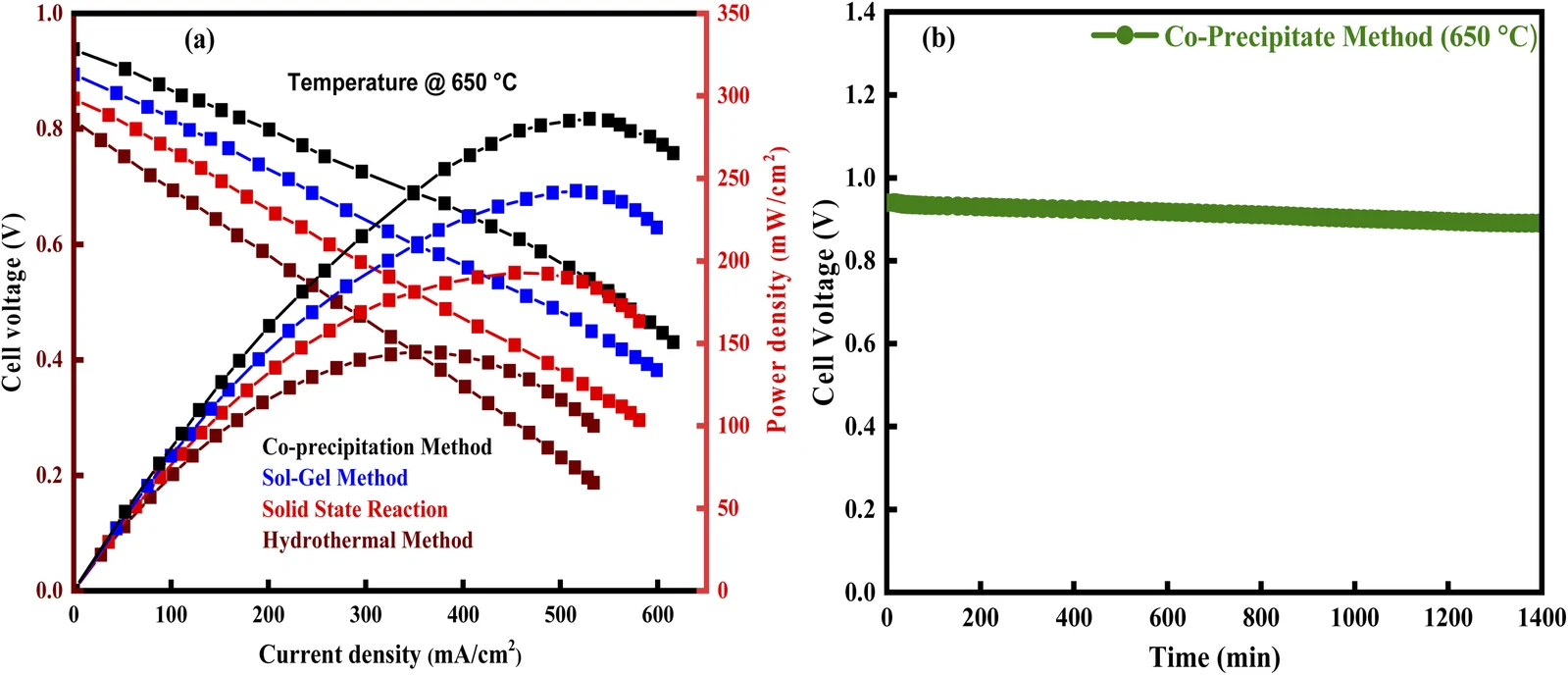
(a) *I*–*V* characteristics showing OCV, current and power densities of fuel cell based upon La_0.63_Ga_0.37_Mo_0.74_Fe_0.26_O_3−δ_ (LGMF) cathode material synthesized by different synthesis methods at 650 °C. (b) Stability test of fuel cell performance based upon La_0.63_Ga_0.37_Mo_0.74_Fe_0.26_O_3−δ_ (LGMF) cathode material for 24 hours.

The high electrochemical efficiency of the La_0.63_Ga_0.37_Mo_0.74_Fe_0.26_O_3−δ_ (LGMF) cathode paired with a Gadolinium-Doped Ceria (GDC) electrolyte relies on synergistic defect chemistry and interface matching. Catalytic mechanism & fuel flexibility: LGMF operates as a Mixed Ionic-Electronic Conducting (MIEC) perovskite matrix. When used symmetrically under reducing fuel conditions (anode side), the transition metal iron Fe^4+^/Fe^3+^ undergoes partial surface reduction, driving oxygen vacancy (Vo) formulation. Under oxidizing conditions (cathode side), the network safely facilitates oxygen reduction reactions (ORR) *via* accelerated surface exchange kinetics. GDC features superior intermediate-temperature ionic conductivity (10^−2^ S cm^−1^. Electronic suppression matrix: The reduction of Ce^4+^ to Ce^3+^ in GDC at high temperatures and reducing atmospheres induces an internal electronic leakage current. The chemical design of the LGMF electrode counterbalances this by creating an optimized structural adhesion that reduces the interfacial Area-Specific Resistance (ASR).

To benchmark the electrochemical performance of the present LGMF-based symmetrical cell, the obtained fuel-cell results have been compared with previously reported symmetrical solid oxide fuel cell systems and prescribed in Table 4.

**Table 4:** Comparison of LGMF cathode material-based fuel cell performance and previous reported symmetrical fuel cell systems

Electrodecomposition	Electrolyte/cellconfiguration	*T* (°C)	OCV(V)	Peak power density (mW cm^−2^)	Reportedresistance (Ω cm^2^)	Fuel/oxidant	Reference
La_0.63_Ga_0.37_Mo_0.74_Fe_0.26_O_3−δ_ (LGMF)	GDC-based configuration	650	0.94	286	0.44 Ω	H_2_/air; humidity not specified in current manuscript	Present study
Sr_2_FeTiO_6−δ_ (SFT)	SDC; SFT/SDC/SFT	800	N/A	335	0.075 (reported cell ASR under wet H_2_)	Wet H_2_/ambient air	36
Ba_0.5_Sr_0.5_Co_0.2_Fe_0.7_Mo_0.1_O_3−δ_ (BSCFM)	GDC buffer layers + YSZ electrolyte; BSCFM/GDC/YSZ/GDC/BSCFM	800	N/A	418	0.035 (interface polarization resistance in H_2_)	Humid H_2_ (3% H_2_O)/static air	37
La_0.6_Ce_0.1_Sr_0.3_Fe_0.95_Ru_0.05_O_3−δ_ (LCSFR)	LSGM; LCSFR-CGO/LSGM/LCSFR-CGO	800	1.07	904	0.11 in air; 0.32 in wet H_2_ (electrode polarization resistance)	Wet H_2_ (3% H_2_O)/air	38
La_0.5_Sr_0.5_Fe_0.9_Mo_0.1_O_3−δ_ (LSFMo)	LSGM; LSFMo/LSGM/LSFMo	850	N/A	640	0.08 in air; 0.09 in wet H_2_ (polarization resistance)	20% H_2_O–H_2_/air	22
Ba_0.5_Sr_0.5_(Co_0.8_Fe_0.2_)_0.8_Mo_0.2_O_3−δ_ (BSCFM)	LSGM; BSCFM/LSGM/BSCFM	800	N/A	1225	0.064 (area-specific polarization resistance)	Wet H_2_ (3% H_2_O)/air	23
La_0.6_Sr_0.4_Fe_0.7_Ni_0.2_Mo_0.1_O_3−δ_	LSFNM-SDCi/DA-SSOFC	800	N/A	487	NR	NH_3_/oxidant	39

### 3.7. Charge transport and electrochemical mechanism of LGMF symmetrical electrodes

The electrochemical performance of the La_0.63_Ga_0.37_Mo_0.74_Fe_0.26_O_3−δ_ (LGMF) cathode material is strongly governed by its defect chemistry and mixed ionic-electronic conduction behavior. Partial substitution of Fe by Mo modifies the local lattice environment and promotes the formation of oxygen vacancies, which plays a critical role in facilitating oxide-ion transport within the perovskite structure. In addition, electronic conduction is supported through Fe^3+^/Fe^4+^ redox hopping, enabling simultaneous transport of electrons and oxide ions during fuel cell operation. The coexistence of ionic and electronic charge carriers enhances the electrochemical activity of the electrode and improves the overall charge transfer process at intermediate temperatures.

Under SOFC operating conditions, oxygen molecules are adsorbed at the cathode surface and reduced to oxide ions through oxygen vacancy sites *via* the oxygen reduction reaction (ORR). The generated O^2−^ ions migrate through the electrolyte toward the fuel electrode, where hydrogen oxidation reaction (HOR) occurs to produce water and release electrons. The released electrons are transported back through the external circuit, completing the electrochemical cycle. Low temperature ceramics fuel cell operating between 400–600 °C temperature planning low materials cost and faster start-up time than the traditional SOFC operating at high temperature in the range of 800–1000 °C focuses to overcome the sluggish ion mobility using semiconductor ceramics materials. The selective materials used in fuel cell components like La_0.63_Ga_0.37_Mo_0.74_Fe_0.26_O_3−δ_ (LGMF) cathode may enhance electrochemical activities, where ionic motion and ion-electron coupled energy flow may evolve from classical diffusion toward resonance-conditioned transport. In the recent research work, synthesis route comparison for La_0.63_Ga_0.37_Mo_0.74_Fe_0.26_O_3−δ_ (LGMF) cathode material indicates that transport descriptions increasingly require attention to state sensitivity, confinement, and interface-conditioned realization, rather than pathway existence alone. This study ensures the structural development in fuel cell cathode materials may empower conductivity and fuel cell performance. The activation energy and area specific resistance ASR curves also depend upon the internal structure of the materials, especially cathode side where electronic and ionic transport mechanisms take place (Fig. 7).^40,41^

**Fig. 7:**
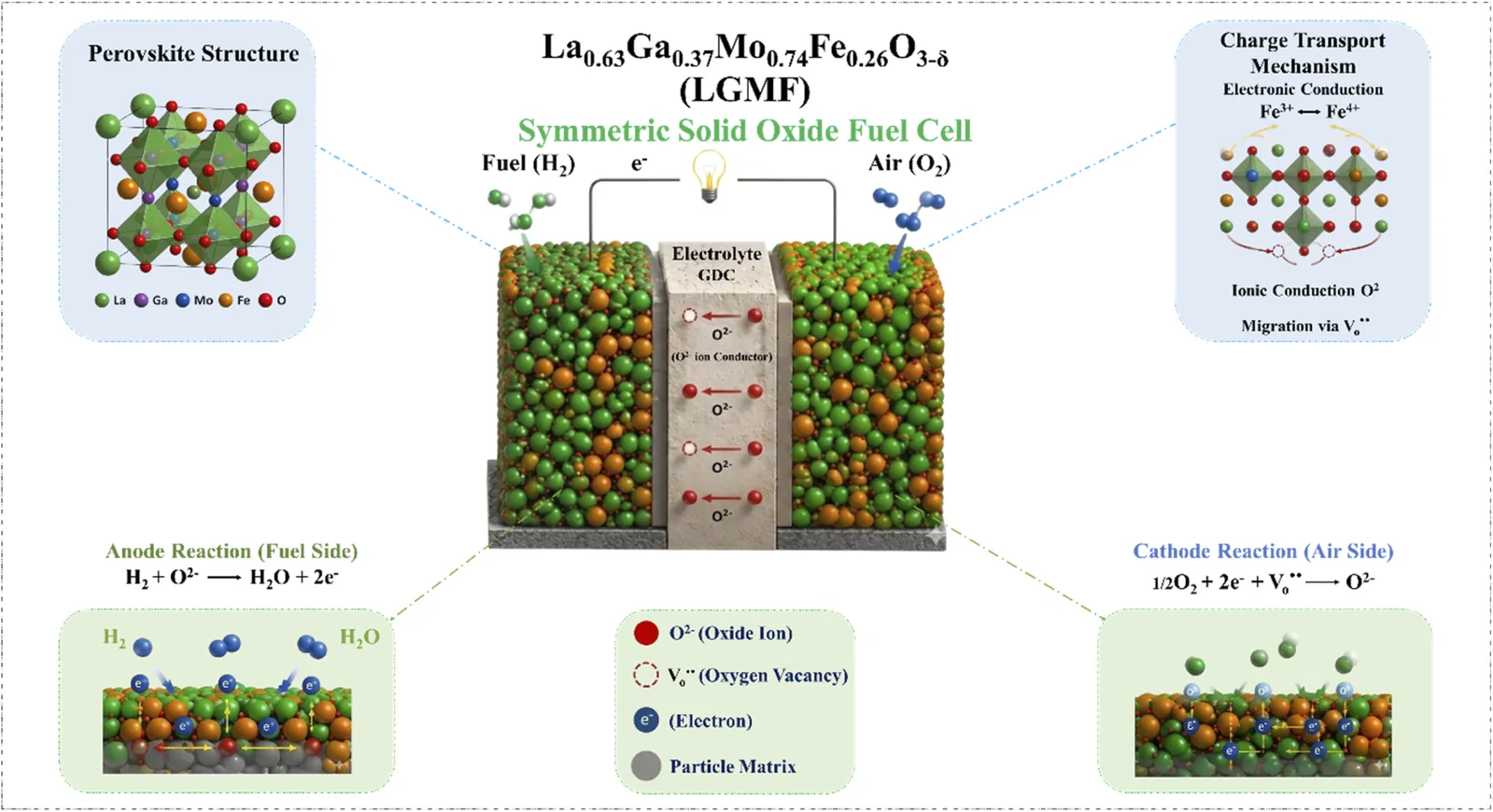
The figure highlights oxygen vacancy formation, mixed ionic electronic conduction, and structure defect electrochemical relationship in La_0.63_Ga_0.37_Mo_0.74_Fe_0.26_O_3−δ_ (LGMF) symmetrical electrodes.

Among all synthesis routes, the co-precipitation method exhibited superior electrochemical performance due to its improved structural homogeneity, reduced grain boundary resistance, and enhanced defect concentration, which collectively contributed to lower ohmic resistance and higher conductivity. The improved microstructure and optimized charge transport pathways obtained through co-precipitation therefore resulted in enhanced electrocatalytic activity and fuel cell performance.

## 4. Conclusion

In this study, the perovskite oxide La_0.63_Ga_0.37_Mo_0.74_Fe_0.26_O_3−δ_ (LGMF) was successfully synthesized *via* four different methods: sol–gel, hydrothermal, co-precipitation and solid-state reaction. The investigation unequivocally demonstrates that the choice of synthesis route has a profound and direct impact on the material's structural, optical and electrochemical properties, which in turn dictates its performance as a symmetrical electrode for IT-SOFCs.

Structural characterization revealed that the co-precipitation and sol–gel methods yielded materials with superior phase purity, crystallinity and a more homogeneous microstructure. These attributes were in stark contrast to the broader peaks and potential secondary phases observed in the solid-state and hydrothermal samples. This structural advantage was further corroborated by optical analysis, where a reduced band gap for the co-precipitated sample indicated a higher concentration of charge-carriers facilitating defect states. The electrochemical performance directly mirrored the structural findings. The co-precipitation method yielded the lowest ohmic resistance (0.44 Ω cm^2^ at 650 °C) and the highest total conductivity (0.70 S cm^−1^ at 650 °C), a result attributed to enhanced ionic-electronic conductivity and optimal particle morphology that minimizes grain boundary resistance. Consequently, when deployed in a symmetrical cell configuration, the electrode synthesized *via* co-precipitation achieved a peak power density of 286 mW cm^−2^, significantly outperforming its counterparts. Therefore, it is concluded that the co-precipitation method is the most promising synthesis strategy for fabricating high-performance LGMF symmetrical electrodes. This method provides an optimal balance of phase purity, defect chemistry and microstructural properties that are critical for exceptional electrocatalytic activity for both oxygen reduction and hydrogen oxidation reactions. This work highlights the critical importance of synthesis route selection in the development of advanced materials for efficient and durable intermediate-temperature solid oxide fuel cells and provides a clear pathway for optimizing electrode performance through tailored fabrication processes.

## Conflicts of interest

There are no conflicts to declare.

## Data Availability

The data is available within the paper.
